# Practical Recommendations for Treatment of Dental Patients with Congenital Bleeding Disorders during the Covid-19 Pandemic: A Narrative Review

**DOI:** 10.3390/ijerph17197245

**Published:** 2020-10-03

**Authors:** Sylwia Czajkowska, Joanna Rupa-Matysek, Lidia Gil, Anna Surdacka

**Affiliations:** 1 Department of Conservative Dentistry and Endodontics, Poznan University of Medical Sciences, Bukowska 70, 60-812 Poznan, Poland; annasurd@ump.edu.pl; 2 Department of Hematology and Bone Marrow Transplantation, Poznan University of Medical Sciences, Szamarzewskiego 84, 60-569 Poznan, Poland; j.rupamatysek@ump.edu.pl (J.R.-M.); lidia.gil@skpp.edu.pl (L.G.)

**Keywords:** COVID-19, severe acute respiratory syndrome coronavirus 2, dentists, blood coagulation disorders, pandemics, cardiovascular system

## Abstract

At the end of 2019, a new disease—COVID-19—was identified, and a few months later, the World Health Organization announced a pandemic. It is now known that SARS-CoV-2 is highly contagious and most confirmed infections are mild to moderate. The situation is particularly difficult for dentists due to the high risk of virus transmission in the dental surgery. Complications including, but not limited to, problems with the respiratory and cardiovascular systems have been reported in patients with SARS-CoV-2. Several changes in the coagulation system, such as lower platelet numbers or increased prothrombin time, as well as increased D-dimer and fibrinogen, were observed. This review is intended to systematize the knowledge on the treatment of patients with congenital bleeding disorders (CBD) during the SARS-CoV-2 pandemic. Extensive literature research was conducted into COVID-19 and the general medical and dental treatment of patients with CBD. Case studies, research results and recommendations of international societies were used in the study. The results of this research are presented in the form of recommendations for the treatment of patients with coagulopathies. It should be remembered that the impact of COVID-19 on the health condition of patients with CBD is unknown to date.

## 1. Introduction

COVID-19 is an acute airway infection caused by severe acute respiratory syndrome coronavirus 2 (SARS-CoV-2) [1]. The first outbreaks were identified at the end of 2019 in Wuhan, Hubei Province, China and, initially, the etiology of the disease was unknown [2]. On 11 March 2020, the World Health Organization (WHO) declared the SARS-CoV-2 pandemic due to the public health threat [3]. The speed at which the virus spreads proves how contagious it is. This is not the first pandemic in human history, but it is the first to cover the whole world. Globalization and the ease of travel are factors that have contributed to the virus’s migration. Based on measurements and recommendations from previous pandemics, many international public health, governmental and other societies have provided guidance which is regularly updated, and several approaches have been implemented in order to control transmission, including reliable monitoring of the SARS-CoV-2 transmission rates and severity, mitigation of the impact of COVID-19 in healthcare and social care settings, detection of clusters or outbreaks in specific settings, and, once achieved, maintenance of COVID-19’s elimination status. The data on previous virus epidemic/pandemic procedures, such as Ebola virus or MERS-CoV (Middle Mast respiratory syndrome coronavirus) and influenza (H1N1 influenza A pandemic or swine flu pandemic), with their severe socio-economic burdens, have been implemented into the current guidelines and recommendations, including those from the WHO and the European Centre for Disease Prevention and Control (EDCD). According to WHO data, as of 11 July 2020, there were 12,322,395 confirmed cases of COVID-19, 556,335 deaths and 216 countries and territories affected [4]. For comparison, another virus from the coronavirus family described in 2012—MERS-CoV, from September 2012—according to WHO data, covered only 27 countries and there were 2494 confirmed cases [5]. Previous pandemics have highlighted the lack of readiness of health professionals to work in a pandemic [6] and the need to provide up-to-date knowledge [7]. Moreover, they have taught medics that in order to prevent epidemics/pandemics, pathogens must be identified quickly, and their transmission should be kept to a minimum. When a new infectious agent is discovered, health authorities assess the risk of blood-borne viral/bacterial transmission, possibly due to the HIV pandemic, in which patients with hemophilia, among others, were inadvertently infected. Based on previous epidemics/pandemics—H1N1, H5N1, SARS, and MERS—we can conclude that there is a low risk of transmission of infection by transfusion [8,9] and a high risk of virus transmission in the dentist’s office due to aerosol formation [10]. In addition, previous pandemics/epidemics, for example the HIV pandemic, have drawn attention to high-risk patients, including CBD patients. Due to the epidemiological situation, healthcare systems around the world are under constant pressure, and the diagnosis and treatment of patients with SARS-CoV-2 has become a priority. As a result, healthcare systems around the world are under constant pressure, and the diagnosis and treatment of patients with SARS-CoV-2 has become a priority. Healthcare facilities are adapting to the current epidemiological situation and it has become a goal and challenge to avoid becoming a source of COVID-19 transmission, which has an impact on the pattern and interdisciplinarity of the treatment of patients with congenital bleeding disorders (CBD), including patients with hemophilia or von Willebrand disease. In truth, the clinical symptoms of infection mimic those of seasonal flu, but the course of the disease can vary greatly, from asymptomatic infections to fatal cases. Those most at risk of increased mortality due to COVID-9 are people with comorbidities and those over the age of 65 [11,12,13,14,15,16]. For this reason, treatment of the underlying disease has become extremely important. In patients with a confirmed SARS-CoV-2 infection, several changes in the coagulation system were observed, which may be related to vascular endothelial damage, inflammatory response with cytokine, activation of complement pathways and neutrophil extracellular traps due to infection [17,18]. The impact of COVID-19 on the health of patients with congenital bleeding disorders (CBD) is still under discussion. The literature suggests the possibility of thromboembolic disorders, also in patients with CBD [19]. There are known cases of bleeding related to COVID-19 infection [20] and also the influence of VWF factor level on the risk of complications is discussed [21,22]. The aim of this article is to give recommendations to doctors and dentists for the optimal management of patients with CBD during the SARS-CoV-2 pandemic.

### 1.1. Normal Hemostasis

Hemostasis, the process of blood clot formation at the site of vessel injury, consists of primary and secondary hemostasis. In primary hemostasis, after endothelia injury and the contraction of the vessel wall, platelets adhere to the collagen through platelet aggregation, secretion and further procoagulant activities leading to a platelet plug which is pivotal for the initial hemostatic response to stop bleeding. Secondary hemostasis causes permanent mechanical closure of the wound with local generation of fibrin and the subsequent formation of a fibrin-based clot based on coagulation factors, which is then followed by repair and scar formation. Initiation of the clotting of secondary hemostasis begins with the generation or exposure of tissue factor at the wound site with activated factor VII, generating a small amount of thrombin which activates factor XI, leading to amplification of thrombin generation in the propagation phase by enzyme complexes (intrinsic tenase and prothrombinase). All these processes reinforce the initial platelet plug and form a clot with a stable fibrin network with activated factor XIII that stabilizes and crosslinks the overlapping fibrin stands [23,24].

### 1.2. Bleeding Diathesis–Pathomechanism and Symptoms

Hemorrhagic disorders can be congenital or acquired and may result from vascular changes, deficiency of clotting factors, or dysfunction or reduction of thrombocytes. The most common inherited bleeding disorder is von Willebrand disease (VWD) [25]. VWD is caused by a quantitative or qualitative disorder of the von Willebrand factor [26]. This factor is essential for the adhesion of thrombocytes to the endothelium at the site of a damaged vessel. This abnormality is mainly related to primary hemostasis. Abnormalities of secondary hemostasis occur, among others, in hemophilia. Hemophilia is a hemorrhagic diathesis in which there is a decrease in factor VIII (hemophilia A), factor IX (hemophilia B) or factor XI (hemophilia C) [27]. In contrast to VWF, hemophilia A and B are an X-linked disease, therefore the diathesis affects only males or homozygous females [28]. Due to the factor deficiency, thrombin generation and clot formation is disturbed with impaired hemostasis and subsequent bleeding complications. The activity of the deficient or residual clotting factor level in the blood correlates with the severity of hemophilia and the severity of the symptoms. Severe (defined as <1 percent factor activity which corresponds to <0.01 IU/mL), moderate (factor activity level ≥1 percent of normal and ≤5 percent of normal) and mild levels of hemophilia (5–40% of the norm) have been distinguished [29]. In patients with hereditary bleeding diathesis, there is mainly excessive or prolonged bleeding, which may even be life threatening (Table 1) [30]. Bleeds that appear include gingival bleeding [31], intra-articular bleeding [31], excessive bleeding after injuries and surgery, for example, after tooth extraction [28,32,33], nasal and oral bleeding [26,31], gastrointestinal bleeding [31], hematomas and bleeding from small wounds [26]. Prolonged bleeding after tooth extraction may also occur in women who are carriers of hemophilia [34]. The cause of bleeding in the oral cavity, in addition to the deficiency of the coagulation factor, may be poor hygiene associated with the fear of possible mechanical injury, thus leading to gingivitis [35]. There are discrepancies in dental health and oral hygiene in individuals with CBD [29,35]. The literature reports a frequent occurrence of osteoporosis in patients with hemophilia [36,37], as well as the possibility of complications related to hemorrhages into muscles and joints [38]. Complications include arthropathy, synovitis, contractures, or the formation of pseudo-tumors within the loco-motor system in the course of hemophilia. In some cases, hemarthrosis may appear, and in rare cases, it may also affect the temporomandibular joint [24]. Pseudo-tumors may occur both in the bones and muscles [39]. In exceptional cases, they are located within the craniofacial bones [40,41,42]. In addition, treatment with plasma-derived concentrates without the appropriate virus inactivation may result in blood-borne infections, including human immunodeficiency virus (HIV), hepatitis B virus (HBV), hepatitis C virus (HCV), human T-cell leukemia virus (HTLV-1), or parvovirus B19 [43].

## 2. Materials and Methods

Criteria for consideration of studies/articles for this review and types of participants.

Extensive literature research including all available studies, case reports and guidelines/recommendations, irrespective of the original language, year or publication status, were included in the analyses if there was sufficient evidence and it was related to the topic. This review is limited to studies related to dental care, dentistry, oral medicine, precautions in dentistry during COVID-19/SARS-CoV-2 or Coronavirus pandemics in patients with CBD, such as hemophilia, von Willebrand disease and other rare bleeding congenital disorders. We used PubMed/Medline for information from the National Library of Medicine with two search strategies; one based on the use of Medical Subject Headings (MeSH) and the other based on text word searches performed by 21 August 2020. If we needed a more focused search, related articles were also evaluated. Also, the recommendations of international societies such as the World Federation of Hemophilia (WFH) and the European Hemophilia Consortium (EHC), European Haemophilia Consortium (EHC) and other societies were investigated. The bibliographic references of all the retrieved studies and reviews were assessed for additional reports of studies. According to the Cochrane Methodology Review Groups, due to the limited number of original articles, no randomised or quasi-randomised studies in this field published before the end date of our search process, the results of this research are presented in the form of recommendations for the treatment of dental patients with congenital bleeding disorders as a narrative review. Obviously, for the oral health of the population, it is impractical to postpone all dental treatment until the pandemic has passed, especially since an end to it is still not in sight. Therefore, the collation of all relevant data should be seen as necessary and ongoing. The aim of our paper was not to segregate data, but rather collate data related to CBD, dental care and precautions during times of pandemics combined with data from previous pandemics. We wished to combine all current knowledge into one paper giving recommendations and guidelines based on the most up-to-date literature available at the moment. Finally, only articles/recommendations in English were investigated. Altogether, 64 articles and 11 websites were included. 

## 3. Results

### 3.1. SARS-CoV-2 Virus

The first cases of infection with severe acute respiratory syndrome-coronavirus 2 were recorded in 2019, in central China. The clinical signs of infection are still being documented and compiled. It is now known that the SARS-CoV-2 infection can vary greatly and includes both asymptomatic infections and pneumonias with high mortality rates [16]. The virus is highly contagious, therefore early diagnosis and isolation of patients with SARS-CoV-2 infection is necessary to control transmission [15]. The incubation period is 2–14 days and virus transmission takes place either directly or indirectly. Contact with aerosol from the airways or saliva is particularly dangerous. In practice, this means that some medical professionals such as dentists and dental hygienists may be at a greater risk of potential infection [44]. The most frequent symptoms appearing in the course of the disease are: fever, dry cough and dyspnea. Less common are diarrhea, nausea, muscle pains, abdominal pain, vomiting, headaches and dizziness [15,16]. Patients with pneumonia caused by the SARS-CoV-2 virus show abnormalities in chest computed tomography [45]. The severity of the disease is influenced by the patient’s age and the presence of comorbidities [11,12,13,14,15,16]. Patients with arterial hypertension, diabetes, cardiovascular diseases, cerebrovascular diseases and/or cancers more often require treatment in the intensive care unit [15,46]. Complications associated with COVID-19 include acute respiratory distress syndrome (ARDS), acute heart injury, acute kidney damage and hepatic impairment [15,16,47]. Moreover, changes have been observed in the clotting system such as disseminated endovascular coagulopathy (DIC) as well as decreased platelet count, prolonged prothrombin time (PT), increased D-dimer levels and increased fibrinogen concentrations [15,17,48,49]. Additionally, there are reports of a possible increase in clotting factor VIII and von Willebrand factor (VWF) levels [17]. This may be related to the damage to the endothelium as a result of COVID-19 as presented in the literature [18]. Decreased factor XII and factor XIII activity has also been described [17]. People who have died of SARS-CoV-2 infection are more likely to have developed systemic inflammation and multi-organ dysfunction when compared to recovered patients [16]. The Chinese Centers for Disease Control classify most of their confirmed COVID-19 cases as mild to moderate [16]. Based on analysis of laboratory tests carried out in China on a group of 138 hospitalized patients, it was found that the most common laboratory abnormalities were prolonged prothrombin time (PT), decreased total lymphocyte count and increased lactate dehydrogenase activity [15]. Moreover, patients treated in the intensive care unit show more deviations in laboratory tests than patients who did not required hospitalization in the intensive care unit—there is an increase in pro-inflammatory cytokines such as interleukin-2 (IL2), interleukin-7 (IL7), interleukin-10 (IL10), tumor necrosis factor α (TNFα), monocyte chemoattractant protein 1 (MCP1), interferon gamma-induced protein 10 (IP10), granulocyte colony-stimulating factor (GCSF) and macrophage inflammatory protein-1 alpha (MIP1A) [15,45].

### 3.2. Congenital Bleeding Diathesis in the Covid-19 Pandemic

Hypercoagulability is a common feature of SARS-CoV-2 infection, but the impact of the infection on CBD patients is still unknown. In the literature, the possibility of thromboembolic disorders in this group of patients, a thrombotic episode in a patient with mild VWF disease [19] and the possibility of bleeding associated with SARS-CoV-2 infection [20] have all been reported. All the while there is a discussion about a possible association between complications and the level of factor VWF [21,22]. It has also been shown that the incidence of concomitant diseases has an impact on the severity and mortality of COVID-19 [11,12,13,14,15,16]. The COVID-19 pandemic is the first pandemic to spread worldwide and knowledge about the virus of SARS-CoV-19 and its impact on patients with CBD is still insufficient. For this reason, patients with CBD require special care and it is extremely important to systematize information.

Patients with CBD, including those with hemophilia, should follow the same preventive measures against SARS-CoV-2 infection as the rest of the population [50,51]. Attention should be paid, among other things, to avoiding touching the face, keeping a minimum distance of 2 m between people, use of strict hand hygiene (soap and water for 20 s), observing cough etiquette, and limiting contact with people [52,53,54]. The WHO, as well as international and national organizations working for patients with bleeding disorders, including the World Federation of Hemophilia (WFH) and the European Hemophilia Consortium (EHC), are monitoring and evaluating the spread of the virus on an ongoing basis, and thus provide the latest recommendations. Based on the collected literature and the recommendations of the WHO, WFH, and EBMT, the authors created the recommendations presented in Table 2.

Patients with CBD and suspicion or diagnosis of SARS-CoV-2 should be mostly quarantined at home—the criteria for admission to hospital should be similar to those for patients without bleeding disorders [50]. It seems important to provide information about the disease to the appropriate associations for patients with CBD. A case of COVID-19 has been reported in a patient with congenital severe hemophilia A. The course of the disease—clinical symptoms and outcomes—did not differ in relation to patients without hemorrhagic diathesis. The patient experienced an elevated body temperature, chills, myalgia, recurrent vomiting, anorexia, a dry cough and dyspnea [62].

Another described case of a patient with a hereditary bleeding disorder and SARS-CoV-2 infection was a woman with factor XIII deficiency. Despite the mild form of COVID-19, the patient developed a hematoma in the abdominal cavity (presumably due to the sudden attacks of coughing), causing bleeding and hospitalization [63]. In order to reduce the risk of possible bleeding into the respiratory tract in a patient with congenital bleeding disorder and infection with SARS-CoV-2 virus, some doctors recommend prophylactic treatment with a concentrate of a deficient agent while maintaining a higher level of clotting factor [52,64]. These bleedings can be caused by increased blood pressure in the brain as a result of increased physical effort, such as coughing or blowing the nose [52]. If a pain reliever and body temperature reduction drug is necessary, paracetamol (acetaminophen) at a dose not exceeding 60 mg/kg/day or 3 mg/day is recommended. Unlike non-steroidal anti-inflammatory drugs, paracetamol does not increase bleeding and does not inhibit the inflammatory response [52]. No contraindications to the use of antivirals or hydroxychloroquine in patients with hemophilia have been reported [50].

If a patient with a CBD is admitted to hospital, it is important to conduct a thorough medical history and to obtain information on possible therapy with emiciuzumab (the effect on coagulation tests based on aPTT) [64], participation in clinical trials (treatment with anti-TFPI antibodies or fitusiran) or gene therapy [50,52]. An essential element of the medical history is to obtain information about other concomitant diseases, as it has been shown that the number of comorbidities correlates with the clinical condition of a patient with SARS-CoV-2 infection. Moreover, comorbidities negatively affect the course of COVID-19 [13]. Hypertension control and daily blood pressure monitoring may reduce the risk of intracranial hemorrhage [50]. If invasive procedures are required during treatment with SARS-CoV-2, it is essential to maintain a high level of coagulation factor in a patient with CBD. This is to prevent any possible bleeding that may occur, for example, during intubation. It is important to protect vessels and not to damage the peripheral veins important for patient home infusion therapy during procedures performed by qualified medical personnel [50]. In the case of home quarantine, it is important to continue with the current therapy and avoid clotting factor deficiencies at home. Patients with hemophilia should have recommended physical activity or self-physiotherapy to prevent loss of muscle mass and joint stiffness [50]. Moreover, exercise is an antithrombotic prophylaxis. SARS-CoV-2 infection has been shown to increase the risk of venous thromboembolism [48]. The impact of SARS-CoV-2 virus infection on patients with CBD is unknown, therefore it is assumed that this group of patients is not protected against thromboembolic or cardiovascular complications. For this reason, patients with a CBD should receive similar anticoagulation therapy to patients without coagulopathy [65,66].

For patients with CBD it is extremely important to continue treatment and to prevent possible complications that could cause hospitalization as patient rotation in the hospital increases the risk of transmission of SARS-CoV-2 virus infection. For this reason, centers treating patients with CBD should expose patients to unchanged amounts of coagulation factors. However, this does not mean that procedures are prohibited in patients with CBD. In each case, the decision on the procedure and possible hospitalization should be made on an individual basis, after assessment of the potential risks and benefits. It is recommended to limit contact with the patient, therefore medical records and telemedical consultation are important [66]. The literature describes the case of a 56-year-old man with a severe form of hemophilia A, who underwent orthopedic surgery despite the difficult epidemic situation. In order to reduce the risk of SARS-CoV-2 infection, the patient was discharged from the hospital after 24 h and the necessary consultations after the surgery were performed via telemedical consultation [67].

As recommended by the World Federation of Hemophilia (WFH) (Table 3), when a patient is treated with recombinant factor VIII or IX with standard or prolonged half-life, emiciumab, FEIBA, factor VIIa, it is not necessary to change the current treatment regimen. For patients treated with plasma-derived factor VIII or IX concentrates, no drug change is recommended as the manufacturing protocols for the inactivation and elimination of viruses are sufficient to eliminate SARS-CoV-2. An individual treatment decision should be made if the patient is using blood products that are not virus inactivated. These products include cryoprecipitate and platelets. The decision should be made after analysis of the potential risks (consequences of potential bleeding if untreated) and benefits. For hemophilia patients participating in clinical trials (except for phase 4 trials), it is recommended that treatment should not be changed or discontinued unless the study team decides otherwise [52].

### 3.3. Dental Treatment of Patients with Congenital Hemorrhagic Diathesis and Covid-19

The SARS-CoV-2 virus is transmitted through airborne contact as well as via symptomatic and asymptomatic patients [68]. The risk of transmission of SARS-CoV-2 virus in the dental surgery is high. This is directly related to the specific nature of the dentist’s work—the long procedures leading to the formation of a close aerosol contact between the dentist and the patient [14,69,70]. Due to the potential risk of infection transmission, failure to prepare dental practices for epidemics as well as fear of infection, the COVID-19 pandemic has had an impact on access to dental clinics and patients’ access to dental services [69,71]. According to recent studies conducted in Beijing during the COVID-19 epidemic, there was an increase in dental and oral infections [71], which can be explained by the abandonment of treatment and a reduction in check-ups. In view of the continuing risk of SARS-CoV-2 infection, national and international dental associations are developing procedures to reduce the risk of virus transmission in the dental practice on an ongoing basis. It is extremely important to monitor country-specific guidelines. The WHO has identified six phases of the pandemic [72]. As each country is in a different phase of the pandemic, it is not possible to define one universal set of guidelines for the whole world. However, it should be remembered that health professionals may be a source of cross transmission [73], so it is crucial to focus on the use of personal protective equipment (including masks with N95/ FFP2 filters or higher), surface disinfection and the application of disposable products where possible [52,55,68,73,74]. The best protection of the respiratory tract is provided by masks with a filter—a mask with an N95 filter provides 8–12 times better protection against particles with a size of 0.04–1.3 micrometers. The symbols N95, N99, N100 refer to the classification from the USA. The European standard defines the filtering parts of the mask as FFP1, FFP2, FFP3. N95 is approximately equal to FFP2 [73]. To this day, no recommendations for the dental treatment of patients with congenital hemorrhagic bleeding convictions during the COVID-19 pandemic have been published. For this reason, general recommendations and guidelines for the treatment of patients with clotting disorders are used in this article.

Prosthetic, periodontal and all other planned procedures are recommended to be postponed until the end of the acute phase of the epidemic [56]. Until then, only emergency patients should be admitted. Urgent situations, as recommended by the American Dental Association (ADA) have been divided into three groups (Table 4) [75]. According to the authors, this classification can be used during the period of the COVID-19 pandemic.

The first category means life-threatening cases, such as uncontrolled bleeding, which may occur, for instance in a patient with congenital hemorrhagic bleeding. In addition, this category includes diffuse inflammatory processes and soft tissue injuries that manifest themselves as intraoral or extraoral edema and may lead to airway obstruction. The above situations require immediate treatment. The second group includes situations that require assistance within 24 h. The aim is to reduce the risk of infection and relieve severe pain. This category includes abscesses and local bacterial infections, pulpitis causing severe pain, pericoronitis (including those related to the difficult eruption of wisdom teeth), post-surgical osteitis and dressing replacement in the case of dry alveolitis, the need to sanitize a cavity before critical medical procedures, biopsy of abnormal tissue, final cementation of a permanent restoration in a situation where a temporary restoration has been broken or lost or irritated the gum, tooth trauma related to partial or complete tooth dislocation, as well as trauma leading to tooth fracture and causing pain or trauma soft tissues. The third category includes procedures that can be performed later than within 24 h. These procedures include the removal of sutures, placement of the prosthesis in oncological patients, correction or repair of the prosthesis in a situation where the functioning is difficult, replacement of the endodontic dressing when pain ailments occur, correction of the wire ligature of an orthodontic apparatus in the event of irritation of soft tissues and ulcer formation, extensive caries, and the presence of defective fillings that cause pain. Any procedures not listed in any category, such as periodic oral examinations or prophylactic treatments, are routine treatments that may be delayed.

In the event of admitting a patient, it is advisable to verify the reasons for contacting the dentist by phone and apply a screening protocol. This is to avoid transmission of the SARS-CoV-2 virus. Information that should be obtained from the patient includes confirmation of a positive SARS-CoV-2 result, contact with a person with a confirmed infection or under quarantine, travel to regions with high COVID-19 incidence, or the presence of symptoms suggesting respiratory disease in patients, including coughing, shortness of breath, and/or an increased body temperature [14,57,68,70]. Despite a negative COVID-19 history, each patient who comes to the dentist’s office should be treated as potentially infected. If a patient with a confirmed diagnosis of COVID-19 comes to the dentist, the patient should not be treated in a normal dental office. It is important to refer the patient to a dental treatment center for patients with confirmed diagnosis of SARS-CoV-2 [57,71]. Pharmacology is an alternative and temporary treatment for emergencies such as swelling or toothache. Treatment with painkillers or antibiotics will allow you to continue treatment after the infection has cleared [68]. As already mentioned, paracetamol (acetaminophen) at a dose not exceeding 60 mg/kg/day or 3 mg/day is the preferred analgesic in patients with CBD [52].

In the event of a patient being admitted by a dentist, in order to reduce the risk of virus transmission in the dentist’s office, it is advisable to instruct the patient to rinse the oral cavity for at least 30 s with disinfectants before any procedures. The solutions used for this purpose are 0.2% chlorhexidine (CHX), 2% listerine, 0.5–1% hydrogen peroxide, 2% iodine povidone [14,57]. It is recommended to minimize the use of rotary and ultrasonic tools as much as possible and replace them with hand tools. Furthermore, it is preferable to work with a rubber dam. It is recommended to position the rubber dam to cover the nose [57]. This is a protection against virus transmission [14,57,68], as well as accidental injury to soft tissues with rotating instruments, which is particularly important in patients with coagulation disorders [58]. It is vital to be especially careful and to carry out procedures, if possible, in an atraumatic manner, this is to prevent possible bleeding. If radiological diagnostics are necessary, preference is given to extraoral examinations, such as conical computed tomography (CBCT) or panoramic radiographs [14,57,68]. Intraoral radiological imaging is not recommended as it may increase saliva secretion, causing coughing or gagging, and consequently increase virus transmission [14,57]. In a situation where it is absolutely necessary to take a 35 mm picture, it is suggested to cover the sensors twice in order to reduce the risk of cross-contamination [68].

It is recommended that prosthetic procedure be postponed until the end of the acute phase of the epidemic [56]. If it is necessary to restore missing teeth with permanent dentures, in patients with congenital hemorrhagic diathesis, supragingival crowns should be considered [59]. Filling missing teeth with removable dentures (partial or complete) has been named as a safe procedure [59,60]. There are no contraindications to orthodontic treatment in this group of patients. However, special attention should be paid to avoiding sharp fragments of wire ligatures [59].

When a patient with a hereditary bleeding disorder develops pain associated with irreversible pulpitis or necrosis, endodontic treatment is a good alternative to extraction and is considered relatively safe in terms of the potential risk of bleeding [58]. Any bleeding that occurs as a result of the presence of living pulp in the canal can be limited by rinsing the root canals with sodium hypochlorite solution [58]. An alternative is a calcium hydroxide dressing, which has drying properties [58,59]. When developing root canals, it is extremely important to observe the working length and use apex locators [58]. This is to prevent the tool from passing through the anatomical opening. The literature describes the use of formocresol in patients with hemophilia in order to mummify the pulp and to control and eliminate bleeding [58]. Local anesthesia often becomes an indispensable element of the dental procedure. It is assumed that infiltration anesthesia administered from the vestibule side of the mouth is safe for patients with hemophilia, and the earlier administration of a deficient coagulation factor has no effect on potential complications [61]. Safe anesthesia in this group of patients also includes pulpal anesthesia, periodontal anesthesia and mental nerve block. However, nerve block anesthesia, administered without prior replacement of the deficient coagulation factor, is associated with an up to 80% risk of hematoma, which may be life-threatening in the event of obliteration of the respiratory tract [59,60]. Infiltration anesthesia administered from the side of the oral cavity is also dangerous due to the presence of a rich network of blood vessels [59,60]. If it is necessary to extract a tooth in a person with a hereditary bleeding disorder, remember about the possibility of excessive or prolonged bleeding [28,32,33]. In some hemorrhagic disorders, such as hemophilia, hemorrhaging may occur several hours or a few days after surgery [32,33]. This is associated with impaired secondary hemostasis, while maintaining efficient primary hemostasis. For this reason, comprehensible delivery of recommendations after surgery is particularly important when treating a patient with congenital hemorrhagic diathesis. It is important to inform the patient about avoiding, in the case of post-extraction pain, painkillers from the group of non-steroidal anti-inflammatory drugs due to their influence on platelet aggregation [32] and to maintain a pressure tampon even 1–2 h after tooth extraction. Treatment of the wound with soluble sutures and dressings, such as TachoComb [59] and tranexamic acid therapy (before and after surgery) seems to be beneficial in preventing secondary bleeding [31,59]. Of course, it should be remembered that preoperative prophylaxis, especially in the case of severe hemorrhagic diathesis, should be discussed with a hematologist.

In the case of patients with congenital deficiency of coagulation factors, dental prophylaxis is of particular importance. Hygiene training, the use of fluoride toothpaste, dental floss and mouth rinses, regular hygiene and dental check-ups are essential to maintain oral health. In addition, prophylaxis allows for the need for dental treatment to be reduced to the minimum and thus has a positive effect on the incidence of emergencies [58].

## 4. Conclusions

The COVID-19 pandemic has had a huge impact on health care facilities and hospitals. The aim is not only to treat patients but also to fight the pandemic. Due to the potential risk of SARS-CoV-2 transmission, doctors and dentists may face dilemmas regarding the diagnosis and treatment of patients, especially those with a heavy medical history. For this reason, it has become extremely important to share knowledge and experiences with other medical professionals. The COVID-19 pandemic is a challenge for both the doctor and the patient, but it can also become an opportunity to integrate the medical community.

## Figures and Tables

**Table 1:** Symptoms of congenital hemorrhagic diathesis.

Congenital Hemorrhagic Diathesis
1. Bleeding gums
2. Hemarthrosis
3. Excessive or prolonged bleeding after injuries
4. Excessive or prolonged bleeding after surgery
5. Bleeding from the nose
6. Bleeding from the mouth
7. Gastrointestinal bleeding
8. Hematomas
9. Bleeding from minor wounds

**Table 2:** Practical recommendations for treatment of dental patients with congenital bleeding disorders during the COVID-19 pandemic.

Before Admitting the Patient [55]
1. Educate the patient (by media, email, etc) about the symptoms of covid-19 and avoiding visits to the doctor’s office if they occur 2. Consider remote patient evaluation 3. Provide a patient-free prescription system 4. Recommend a patient with mild covid-19 symptoms to follow home quarantine 5. Inform a patient with severe covid-19 symptoms about access to hospital help 6. Follow procedures for people with covid-19 symptoms for a patient living with someone with covid-19 symptoms
**During a Patient Visit [55]**
1. Screening and triaged Screen everyone at the first point of contact with a healthcare facility to reduce transmission of the virus 2. Hand hygiene Should be done in accordance with WHO’s My 5 Moments for Hand Hygiene Prepare in accordance with a preparation based on alcohol containing at least 70% alcohol or water with soap Always wash hands before touching the patient Always wash hands before performing a clean/aseptic procedure Always wash hands after exposure to body fluid Always wash hands after touching the patient or his surroundings 3. Respiratory hygiene Provide any patient with suspected COVID-19 a medical mask Adhere to the cough label Remember hand hygiene after contact with objects that may be contaminated with secretions from the respiratory tract or contact with secretions from the respiratory tract 4. Use of personal protective equipment Wear FFP2, FFP3, N95, N99 respirator (or medical face mask if there is a shortage of respirators) Wear gloves Wear goggles or a face shield Consider using long-sleeved medical clothing 5. Use a physical system (1.5–2m) or have only one patient in the waiting room [52,53,55]
**Surface Disinfection [55]**
1. Clean surfaces with water and detergent 2. The recommended disinfecting solution is 0.1% (1000 ppm) sodium hypochlorite or 70–90% ethanol 3. In case of contact of the surface with blood or body fluid, the recommended concentration of sodium hypochlorite is 0.5% (5000 ppm) 4. The recommended contact time of disinfectants on the base of the above-mentioned substance is at least 1 min
**Surgical Procedures [55]**
1. Base surgical decisions on the need, risks and benefits of the procedure, and the patient’s clinical condition. 2. Do not base decisions on covid-19 status 3. Take into account the patient’ clinical condition, the need for surgery, and the benefits and risks of the procedure 4. It should be considered whether non-surgical treatment might be an alternative 5. Conduct a risk assessment if the procedure cannot be postponed 6. Perform a molecular test on samples from the upper respiratory tract in patients with symptoms and suspected COVID-19 7. Do not delay urgent surgery if it is not possible to perform the above-mentioned test 8. Wear a surgical mask when transporting a patient with COVID-19 to the operating room
**Home Quarantine of a Patient with CBD [52]**
1. The criteria for admission to hospital are similar to those of patients without CBD 2. Prevent deficiency of clotting factors at home 3. Recommend physical activity or self-therapy 4. Limit contact with the patient—use telemedicine
**Hospitalization of a Patient with CBD [52]**
1. Hospital contact with HTC 2. Provide replacement therapy and/or venous access 3. Get information on replacement therapy Pay special attention if the patient is being treated with emicizumab, has undergone gene therapy or is in a medical experiment with rebalancing agents (anti-TFPI and fitusiran) 4. Get information on comorbidities 5. Consider maintaining a higher clotting factor level 6. Control blood pressure to lower the risk of intracranial hemorrhage 7. Paracetamol is the preferred analgesic and antipyretic drug 60 mg/kg/day or 3 g/day 8. Up-to-date knowledge
**Dental Procedures—Patient with CBD**
1. Refer a patient with COVID-19 symptoms in need of urgent dental care to a designated facility [54] 2. Recommend the patient to visit the office by appointment and plan it in a way to minimize contact between patients 3. Discourage the patient from coming to appointments with an accompanying person, unless it is necessary [54] 4. Postpone all planned procedures, including prosthetic and periodontal procedures, until the end of the acute phase of the epidemic [56] 5. Instruct the patient to rinse the mouth before the procedure for at least 30 s with disinfectants (e.g., 0.2% chlorhexidine (ChX), 2% listerine, 0.5–1% hydrogen peroxide, 2% iodine povidone) [14,57] 6. Minimize the use of rotary and ultrasonic tools [57] 7. Implementation of a rubber-dam is recommended [57] 8. Preference is given to extraoral examinations Cover the sensors twice to reduce the risk of cross-contamination when the taking of an endo-oral x-ray film is absolutely necessary [14,57] 9. Endodontic treatment is preferable to extraction [58] 10. Safe local anesthesia [59,60,61] infiltration anesthesia from the side of the oral vestibule pulpal anesthesia periodontal anesthesia mental nerve block

**Table 3:** Modified recommendations by World Federation of Hemophilia (WFH).

Standard or Extended Recombinant Half-life FVIII/FIX Concentrates, FEIBA, FVIIa, Emicizumab
1. Not recommended to change product
**Plasma-Derived FVIII/FIX**
1. Not recommended to change product
2. Viral inactivation and elimination procedures are sufficient to destroy SARS-CoV-2
3. Blood-derived products not to be virally inactivated (e.g., cryoprecipitate, platelets)—treatment decisions should be based on risk and benefit analyses
**Clinical Trials**
1. Continue treatment if you are currently receiving clinical trial treatment unless the study team decides otherwise
2. If you are not receiving clinical trial treatment discuss postponement with the study team

**Table 4:** Modified dental emergency by American Dental Association (ADA).

Dental Emergencies
1. Uncontrolled bleeding
2. Cellulitis or a diffuse soft tissue bacterial infection with swelling that potentially compromises the patient’s airway
3. Trauma involving facial bones, potentially compromising the patient’s airway
**Urgent Dental Care**
1. Pulpitis with severe pain
2. Pericoronitis or third-molar pain
3. Surgical post-operative osteitis, dry socket
4. Abscess/localized bacterial infection resulting in localized pain, swelling
5. Pain and swelling
6. Dental trauma with avulsion/luxation tooth fracture resulting in pain or causing soft tissue trauma
7. Dental treatment prior to critical medical procedures
8. Biopsy of tissue
9. Crown/bridge cementation if a temporary restoration is lost/ broken/ causing gingival irritation
**Other Urgent Dental Care**
1. Pain due to deep caries or loss of filling
2. Suture removal
3. Denture adjustment or repairs: on radiation/oncology patients when function impeded
4. Replacement of a temporary filling during endodontic treatment in patients experiencing pain
5. Fitting/shortening of an orthodontic wire where it irritates the gums
